# Project ECHO: A Telementoring Program for Cervical Cancer Prevention and Treatment in Low-Resource Settings

**DOI:** 10.1200/JGO.2016.005504

**Published:** 2016-10-05

**Authors:** Melissa S. Lopez, Ellen S. Baker, Andrea M. Milbourne, Rose M. Gowen, Ana M. Rodriguez, Cesaltina Lorenzoni, Catherine Mwaba, Susan Citonje Msadabwe, José Humberto Tavares, Georgia Fontes-Cintra, Gustavo Zucca-Matthes, Donato Callegaro-Filho, Danielle Ramos-Martin, Icaro Thiago de Carvalho, Robson Coelho, Renato Moretti Marques, Thiago Chulam, Mila Pontremoli-Salcedo, Fernanda Nozar, Veronica Fiol, Mauricio Maza, Sanjeev Arora, Ernest T. Hawk, Kathleen M. Schmeler

**Affiliations:** **Melissa S. Lopez**, **Ellen S. Baker**, **Andrea M. Milbourne**, **Ernest T. Hawk**, and **Kathleen M. Schmeler**, The University of Texas MD Anderson Cancer Center, Houston; **Rose M. Gowen**, Su Clinica Familiar, Brownsville; **Ana M. Rodriguez**, The University of Texas Medical Branch, Galveston, TX; **Cesaltina Lorenzoni**, Hospital Central de Maputo, Maputo, Mozambique; **Catherine Mwaba**, **Susan Citonje Msadabwe**, Cancer Diseases Hospital, Lusaka, Zambia; **José Humberto Tavares**, **Georgia Fontes-Cintra**, **Gustavo Zucca-Matthes**, and **Robson Coelho**, Hospital de Cancer de Barretos, Barretos; **Donato Callegaro-Filho**, **Danielle Ramos-Martin**, **Icaro Thiago de Carvalho**, and **Renato Moretti Marques**, Hospital Israelita Albert Einstein; **Thiago Chulam**, A.C. Camargo Cancer Center, Sao Paulo; **Mila Pontremoli-Salcedo**, Federal University of Health Sciences/Santa Casa de Misericordia, Porto Alegre, Brazil; **Fernanda Nozar** and **Veronica Fiol**, Universidad de la Republica, Montevideo, Uruguay; **Mauricio Maza**, Basic Health International, San Salvador, El Salvador; and **Sanjeev Arora**, University of New Mexico, Albuquerque, NM.

## Abstract

Cervical cancer incidence and mortality rates are significantly higher in low- and middle-income countries compared with the United States and other developed countries. This disparity is caused by decreased access to screening, often coupled with low numbers of trained providers offering cancer prevention and treatment services. However, similar disparities are also found in underserved areas of the United States, such as the Texas-Mexico border, where cervical cancer mortality rates are 30% higher than in the rest of Texas. To address these issues, we have adopted the Project ECHO (Extension for Community Healthcare Outcomes) program, a low-cost telementoring model previously proven to be successful in increasing local capacity, improving patient management skills, and ultimately improving patient outcomes in rural and underserved areas. We use the Project ECHO model to educate local providers in the management of cervical dysplasia in a low-resource region of Texas and have adapted it to inform strategies for the management of advanced cervical and breast cancer in Latin America and sub-Saharan Africa. This innovative approach, using ECHO, is part of a larger strategy to enhance clinical skills and develop collaborative projects between academic centers and partners in low-resource regions.

## INTRODUCTION

Cancer is one of the leading causes of death worldwide. In 2012, approximately 14 million new cases and 8.2 million cancer-related deaths were reported.^1^ Cancer and other noncommunicable diseases are responsible for > 60% of deaths globally. In low- and middle-income countries (LMICs), cancer is a primary cause of early death, and its prevalence has been increasing steadily, partly because of population aging and improved control of infectious diseases.^2,3^

Cervical cancer is a notable example of cancer disparities in LMICs. Worldwide, cervical cancer is the fifth most common cancer among women.^1^ However, in the United States, cervical cancer is now relatively uncommon; it is the 11th most common cancer among women.^4^ The development of the Papanicolaou (Pap) test and the introduction of organized screening programs have led to a 70% decrease in cervical cancer incidence and mortality rates over the past 60 years in the United States and other high-income countries.^4^ In contrast, cervical cancer remains the second most common cancer among women in LMICs, and it is the most common cancer in many regions of sub-Saharan Africa.^5^ For example, in Mozambique, cervical cancer is the most common cancer in women, followed by breast cancer.^6^ Of note, higher rates of cervical cancer are also seen in medically underserved areas of the United States because of a lack of regular screening and limited access to care.^7^ Once such area is the Rio Grande Valley (RGV) of south Texas, located along the Texas-Mexico border. The population in this area is largely Hispanic and is medically underserved. The cervical cancer incidence and mortality rates in this region are 30% higher than in the rest of Texas.^8^

There are many reasons for higher cervical cancer rates in LMICs and underserved regions of the United States. These populations are less likely to receive cervical cancer screening because of economic, social, educational, and geographical barriers. In addition, there is often a shortage of locally available trained providers to perform screening tests and to manage patients with abnormal findings according to evidence-based guidelines, including performing colposcopy, cervical biopsies, and loop electrosurgical excision procedures. Furthermore, many women with abnormal screening tests do not receive the recommended diagnostic and treatment procedures because they are unable to travel to central health care facilities for the multiple necessary follow-up visits because of the long distances and high costs associated with travel. Thus, increased participation in screening, together with navigation services and an extension of diagnostic and treatment services, is needed to decrease cervical cancer rates in underserved areas worldwide.

## SPECIFIC CHALLENGES RELATED TO TRAINING AND EDUCATION IN LMICS AND LOW-RESOURCE REGIONS IN THE UNITED STATES

The number of trained physicians and nurses in LMICs is extremely low in comparison with high-income countries. For example, there are 2.6 physicians in Mozambique per 100,000 population compared with 247 in the United States and 222 in the United Kingdom for the same population. Furthermore, few of the physicians in LMICs have specialty training and are capable of treating the high volume of patients presenting with cancer.^9^ Shortages of clinicians, including specialists, are also found in the RGV area of Texas, a medically underserved region. In the RGV, there are currently no local public hospitals, and there are 40% fewer physicians and 50% fewer nurse practitioners per 100,000 people compared with the Texas average.^10^ Project ECHO (Extension for Community Healthcare Outcomes), a telementoring program, can help increase clinical capacity in such low-resource settings.

## PROJECT ECHO TELEMENTORING

Project ECHO was developed in 2003 by Sanjeev Arora, MD, a hepatologist at the University of New Mexico (UNM), to improve both provider capacity and access to specialty care for rural and underserved populations.^11,12^ ECHO is a low-cost, high-impact initiative linking multidisciplinary specialist teams with community primary care clinicians through regularly scheduled teleECHO clinics, in which the participants use videoconferencing to comanage patient cases, and specialists share their expertise via mentoring, guidance, feedback, and didactic education. This approach has enabled clinicians in medically underserved areas to develop the skills, confidence, and knowledge to treat patients with common, complex diseases in their own communities, thereby reducing travel costs, wait times, and avoidable complications. Project ECHO is different from telemedicine, in which the specialist assumes the care of the patient, but instead, involves telementoring, in which the community clinician retains responsibility for managing the patient, operating with increasing independence as his/her skills and self-efficacy grow. Clinicians in underserved areas learn from the university specialists and from each other, and specialists learn from the community providers. This is a many-to-many approach, as opposed to the approach of traditional telemedicine.

The first teleECHO clinic at UNM was developed for the management of patients with hepatitis C virus (HCV) in rural New Mexico.^13^ Providers from 16 rural community clinics and five prisons throughout New Mexico participated in weekly HCV teleECHO clinics with specialists from UNM, presenting their cases, including patients' medical histories, laboratory results, treatment plans, and individual challenges, and asked questions and received guidance about best practices. Specialists from the fields of hepatology, infectious diseases, psychiatry, and pharmacology at UNM provided advice and clinical mentoring during these teleECHO clinics. Working together, the community providers and specialists managed the patients' care according to evidence-based guidelines. The effectiveness of the HCV ECHO clinic was evaluated in a prospective cohort study of 407 patients with chronic HCV that was published in *New England Journal of Medicine* in 2011.^11^ This study compared the outcomes of patients treated by specialists at UNM with those of patients treated by primary care providers at the 21 rural ECHO clinics. There were no significant differences in sustained viral response between the UNM cohort (57.5%) and the ECHO cohort (58.2%). Furthermore, serious adverse events were higher in the UNM cohort (13.7%) than in the ECHO cohort (6.9%). Specifically, Project ECHO improved patient satisfaction, physician self-efficacy, and patient outcomes while concomitantly reducing regional disparities in evidence-based HCV management across the state of New Mexico.

Project ECHO has since expanded to cover almost 50 other specialty areas across the United States and globally.^14,15^ TeleECHO clinics are currently conducted at 82 hub institutions in 13 countries for the management of conditions such as cancer, addictions, rheumatology, HIV/AIDS, dementia, palliative care, autism, diabetes, and cardiovascular disease worldwide.

## PROJECT ECHO AT THE UNIVERSITY OF TEXAS MD ANDERSON CANCER CENTER, UNITED STATES

**Fig 1:**
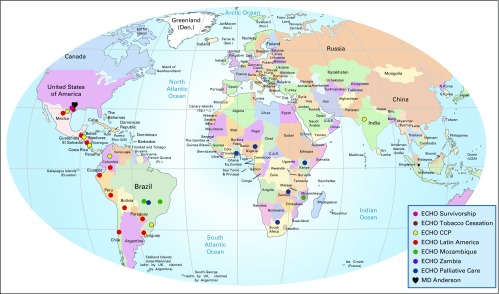
The University of Texas MD Anderson Cancer Center’s Project ECHO programs. ECHO, Extension for Community Healthcare Outcomes. *This program is under development and some of the locations might change in the future; †this program is currently under early discussions with sister institutions in North America.

Our group recently adopted Project ECHO for cancer prevention and management. The Cervical Cancer Prevention Project ECHO clinics are held via a free videoconferencing platform for 1 hour, every other week, at a time convenient for the providers (before their clinics start). Continuing medical and nursing education credits are awarded, free of charge, after each session, and providers receive direct input on case management. The first 45 minutes involve case discussions. Case details (without patient-identifying information) are sent to the specialists by the providers before each ECHO clinic and are presented to the group during the videoconference. In general, an interactive and lively discussion follows the case presentations. Case discussions are followed by a 15-minute didactic presentation by a participating faculty member or a guest lecturer. A few minutes are reserved at the end of the session for additional questions or comments from the participants. The ECHO programs at MD Anderson started as an initiative for the RGV and have since been expanded globally to other low-resource areas (Fig 1).

## PROJECT ECHO FOR THE PREVENTION AND TREATMENT OF CERVICAL CANCER IN THE RGV

The University of Texas MD Anderson Cancer Center’s first Project ECHO program is run in collaboration with the University of Texas Medical Branch (UTMB), the University of Texas Health Science Center School of Public Health Brownsville Regional Campus, and Su Clinica Familiar, a federally qualified health center in the RGV area of south Texas (Table 1). In this region, cervical cancer incidence and mortality is 30% higher than in the rest of the state, and there is a significant shortage of providers and specialists. The US Census Bureau estimated the population in this region to be 1,336,323 in 2014.^16^ Approximately 90% of the population is Hispanic (mostly Mexican American), and approximately 35% of the population lives below the federal poverty line. Seventy percent do not have health insurance, and among those who do, two thirds have Medicare or Medicaid.^17,18^

**Table 1:**
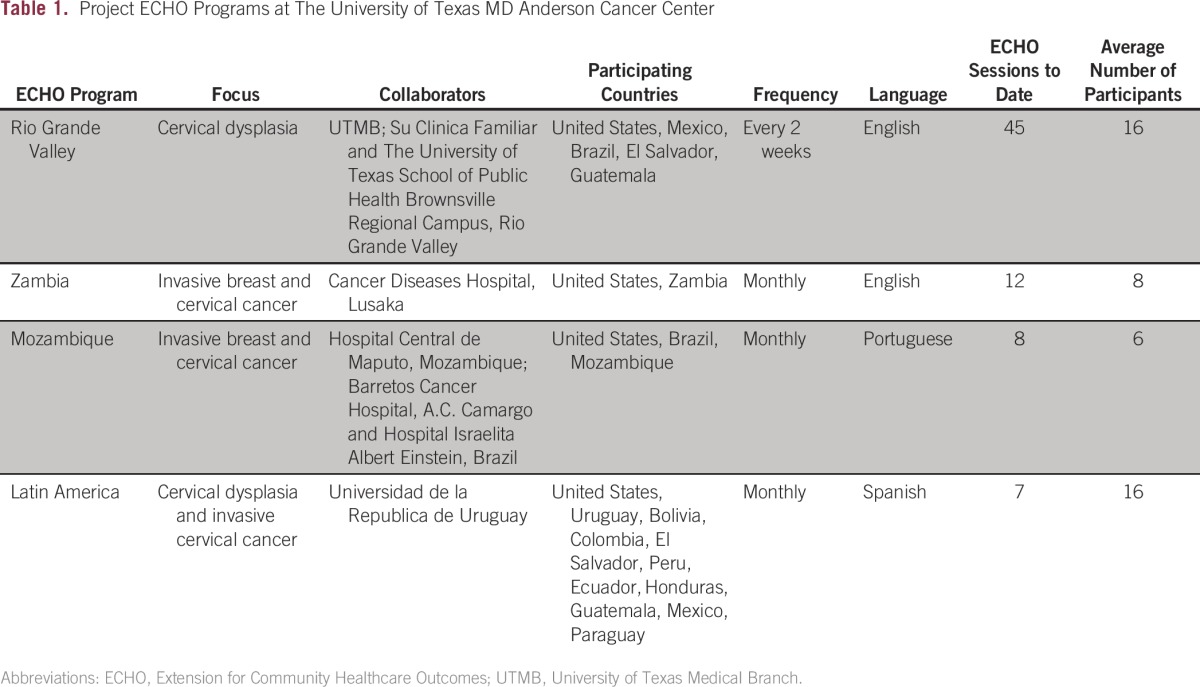
Project ECHO Programs at The University of Texas MD Anderson Cancer Center

The Project ECHO program started in April 2014 as part of a larger strategy with the aims of increasing professional capacity in the RGV community and increasing public participation in regular cervical cancer screening. To increase cervical cancer screening in this region, community health workers use an evidence-based approach to educate women about the importance of screening and human papillomavirus vaccination in combination with patient navigation services. In parallel, Project ECHO is improving health care provider skills in managing abnormal cervical cancer screening tests using existing evidence-based guidelines. Hands-on training complements this Project ECHO initiative. Five local providers have been trained to perform colposcopy through the American Society for Colposcopy and Cervical Pathology course and mentoring program, which requires the participant to perform colposcopic procedures and cervical biopsies under the direct supervision of a mentor. This is accomplished by faculty from MD Anderson and UTMB serving as the mentors and traveling regularly to the RGV area for hands-on training and supervision. Participants from the RGV travel to MD Anderson and partner hospitals for additional training. In addition to the five providers trained in colposcopy, a provider has been trained to perform loop electrosurgical excision procedures, which allows more patients in the RGV to receive treatment locally without the need for referral to a distant facility.

The RGV Cervical Cancer Prevention ECHO program has since expanded and currently includes clinicians from Mexico, El Salvador, Colombia, and Brazil who are interested in cervical cancer prevention. The multidisciplinary specialist team from MD Anderson and UTMB provide input and guidance for patient management and program operations. In the first 2 years of Project ECHO implementation, 45 videoconference sessions have been held, with an average of 16 providers per session, including gynecologists, family physicians, nurse practitioners, physician assistants, and midwives. A preliminary survey of provider satisfaction after 1 year of the program suggests that the majority of providers find the clinics useful in patient management and in improving their skills and knowledge.

## PROJECT ECHO LATIN AMERICA

In 2015, a Project ECHO program for cervical cancer prevention and management was created for providers practicing in Latin America. This program was initiated by providers from Uruguay who were participating in the ECHO Program for the RGV and wished to expand the program to their colleagues in Latin America and to hold the videoconferences in Spanish. The sessions are comoderated by gynecologic oncologists and gynecologists from MD Anderson, UTMB, and the Universidad de La República in Uruguay. To date, seven sessions have been held, with an average of 18 participants from various institutions in Mexico, El Salvador, Guatemala, Colombia, Bolivia, Paraguay, Ecuador, Peru, Uruguay, Chile, and Brazil (Table 1). Because the landscape of cancer screening and treatment is so diverse in Latin America, this forum offers the opportunity to share cervical cancer prevention and treatment experience, with the aim of closing the gap of knowledge and decreasing disparities.

## PROJECT ECHO AFRICA

We have expanded the Project ECHO program by partnering with clinicians in Zambia and Mozambique, specifically for the management of cervical and breast cancer. Project ECHO Zambia is conducted in collaboration with physicians and nurses from the Cancer Diseases Hospital in Lusaka. These videoconferences are held monthly, with the focus alternating between breast cancer and cervical cancer. To date, 12 sessions have been held, with an average of eight participants per session including gynecologists, radiation oncologists, medical oncologists, surgeons, nurses, radiation therapists, medical physicists, and palliative care specialists from MD Anderson and medical oncologists/radiation oncologists, a surgeon, nurses, and physicians from Zambia (Table 1). These videoconferences are complemented by physician and provider exchanges, workshops, and hands-on training sessions. The Zambian team visited MD Anderson twice in 2015, and the MD Anderson team visited Zambia once in 2015 and once in 2016.

Project ECHO for the management of cervical and breast cancer in Mozambique includes clinicians from the Hospital Central de Maputo. This project is a multicenter partnership between the MD Anderson Cancer Center and three MD Anderson sister institutions in Brazil (Barretos Cancer Hospital, Hospital Israelita Albert Einstein, and A.C. Camargo Cancer Center). These teleconferences are held in Portuguese, with a multidisciplinary team of specialists present at each session. To date, nine sessions have been held, with an average of six attendees per session including gynecologists, radiation oncologists, medical oncologists, surgeons, and fellows (Table 1). This program is complemented by hands-on training; in January 2015, 10 physicians, including specialists in breast surgical oncology, pediatric oncology, head and neck surgery, gynecologic oncology, medical oncology, and radiation therapy, visited Maputo, Mozambique, to deliver surgical and clinical training for these different specialties. Clinicians from Mozambique participated in training sessions in Brazil in April 2016. An ongoing relationship is supported by the ECHO infrastructure.

## CHALLENGES OF USING THE ECHO MODEL IN CANCER PREVENTION AND MANAGEMENT

The Project ECHO model was created for the treatment of a common infectious disease, with clear metrics for treatment success identifiable in a short period of time. Although cervical dysplasia is common in the United States, cervical cancer is relatively rare. In addition, the time of progression from dysplasia to cancer may be > 10 years, so individual patient outcomes are not an effective way to monitor program success. Cervical cancer is a progressive disease requiring different types of providers as the disease progresses from dysplasia to cancer. Complicating this is the fact that there are different approaches to care within regions and across borders that may vary according to differences in health policies, standards of care, and resources. Providers screening for cervical cancer are frequently not the same providers treating dysplasia and invasive disease. Furthermore, there is often limited communication between gynecologic oncologists treating cervical cancer and providers performing cervical cancer screening. In LMICs, these communication challenges also exist, complicated by the lack of accurate record keeping, the difficulty in tracking patients, and the lack of specialty providers, such as gynecologic oncologists. These conditions create an opportunity in particular for the Project ECHO program in Latin America, because this ECHO program looks to provide a comprehensive approach to the natural history of cervical cancer (discussions alternate between cervical cancer prevention and cervical cancer treatment). Providers engaged in screening and early detection strategies interact with oncologists treating patients with invasive cancer during these videoconferences.

The MD Anderson ECHO programs, in particular those engaged with international partners in LMICs, must also consider the local context and local resources in care delivery. Attaining the standard of care in the United States is not feasible in many regions, and, given limited resources, creative solutions for providing basic services provide a basis for many discussions. In addition to resource limitations, cultural differences and difficulty initiating change can create challenges and often require unique, region-specific strategies for care delivery. Regular videoconferences help build trust and encourage the development of partnerships through the exchange of information and knowledge.

Project ECHO videoconferences require Internet connections, and in some regions, this is a major challenge. We have developed some alternative strategies including additional phone connections if the Internet connection is unstable; however, this remains an issue for some partners.

## METRICS AND EVALUATION

The evaluation of the programs is ongoing and includes three components: process metrics, provider satisfaction, and levels of collaborations. The process metrics include the number of participants, the number of ECHO sessions held, and the number of individual cases discussed. Provider satisfaction and self-efficacy are measured at baseline and will continue to be measured with follow-up surveys once a year. Furthermore, collaborative efforts will be measured through the number of workshops delivered successfully; the number of joint research programs; the number of providers participating in colposcopy, surgery, and other workshops; and the number of observerships and trainee exchanges completed. In addition, a parallel effort in the RGV is evaluating the impact of Project ECHO and related programs by measuring changes in the number of women undergoing cervical cancer screening and receiving appropriate management of abnormal results, as well as in the number of women diagnosed with high-grade cervical dysplasia and invasive cancer.

## NEXT STEPS

In the short term, the program is engaging additional providers working in medically underserved areas such as other Texas-Mexico border areas and other LMICs. Existing programs are also expanding to include initiatives in other cancer types as well as palliative care. Furthermore, we are developing additional hands-on training programs, workshops, and observerships. Our long-term goal is to continue to provide training, mentoring, and support for providers in medically underserved areas to substantially reduce the incidence of cervical cancer and other malignancies and provide optimal care for patients with these diseases.
